# MicroRNA Mechanisms of Action: What have We Learned from Mice?

**DOI:** 10.3389/fgene.2015.00328

**Published:** 2015-11-16

**Authors:** Hyun Yong Jin, Changchun Xiao

**Affiliations:** ^1^Department of Immunology and Microbial Science, The Scripps Research InstituteLa Jolla, CA, USA; ^2^Kellogg School of Science and Technology, The Scripps Research InstituteLa Jolla, CA, USA

**Keywords:** microRNA mechanism of action, translation regulation, mRNA degradation, miR-430, transient transfection of miRNA mimics, miRNA mutant mice

MicroRNAs (miRNAs) are endogenously encoded single-stranded RNAs of about 22 nucleotides (nts) in length that play essential roles in a large variety of physiological processes in animals and plants (Ambros, 2004; Bushati and Cohen, 2007). Mature miRNAs are integrated into the *R*NA-*i*nduced *s*ilencing *c*omplex (RISC), whose core component is one of the Argonaute family proteins. MiRNAs then direct RISCs to target mRNAs, which are recognized through partial sequence complementarity. Bioinformatic prediction and experimental target gene identification have shown that a miRNA binds mRNAs of hundreds of protein coding genes, which often span a broad spectrum of functional categories (Bartel, 2009; Chi et al., 2009; Hafner et al., 2010). The functional consequence of miRNA-target mRNA interaction and the mechanism of miRNA action have been under intensive investigation and remain a matter of hot debate. It was initially thought that miRNAs repress the protein output of a small number of target genes without significantly affecting their mRNA levels in animals (Lee et al., 1993; Wightman et al., 1993). Subsequent genetic studies in *C. elegans* and zebrafish showed that miRNAs promote the degradation of their target mRNAs (Bagga et al., 2005; Giraldez et al., 2006). Later, a series of genome-wide studies of *in vitro* cultured mammalian cell lines transiently transfected with chemically synthesized miRNA mimics led to the conclusion that the predominant functional consequence of miRNA action is target mRNA degradation (Guo et al., 2010). A follow-up study employing temporal dissection of zebrafish development seems to reconcile these two opposite observations by revealing that translational repression precedes target mRNA decay, and suggesting that the immediate outcome of miRNA-target mRNA interaction is translation inhibition but mRNA degradation can follow (Bazzini et al., 2012). Similarly, re-analysis of the previous datasets from cultured cell lines transiently transfected with synthetic miRNA mimics also found that translation repression precedes mRNA degradation (Larsson and Nadon, 2013).

However, the model miRNA used in the aforementioned zebrafish study, miR-430, is unique in that its expression is rapidly induced and reaches millions of copies per cell in a few hours after fertilization. This expression level of miR-430 is at least 10 times more than all mature miRNAs combined in a mammalian cell, and serves the single purpose of degrading its target genes, maternal mRNAs, at the maternal-zygotic transition (Giraldez et al., 2006). Mammalian cells often express 100–200 different species of miRNAs (Kuchen et al., 2010), with a total amount of 1–2 × 10^5^ copies of mature miRNAs in a cell (Calabrese et al., 2007; Janas et al., 2012). The most abundant miRNAs are often expressed at the level of ~2 × 10^4^ copies per cell (Neilson et al., 2007; Kuchen et al., 2010). As an extreme example, miR-122 is expressed at the estimated level of 5 × 10^4^ copies per cell in hepatocytes (Chang et al., 2004; Jopling et al., 2005). This is still about 20 times lower than the million-copy-per-cell expression level of miR-430 in zebrafish embryos. Considering that the estimated copy number of Argonaute proteins in a mammalian cell is of the same order of magnitude as the total amount of mature miRNAs (1.5 × 10^4^–1.7 × 10^5^; Janas et al., 2012; Wang et al., 2012), the million-copy-per-cell expression level of miR-430 is unlikely to be physiologically relevant in mammalian cells. Therefore, the *in vivo* mechanism of action of mammalian miRNAs remains to be a central question in the field of miRNA research.

In contrast to these desperate efforts to search for a unified model of miRNA mechanism of action, studies of individual functional targets in primary cells or tissues from miRNA mutant mice are painting a rather different picture. Depending on miRNAs, target genes, and cellular contexts, the outcome of miRNA-target mRNA interactions could be predominantly translation repression or mRNA degradation, or a mixture of both. This heterogeneity in miRNA mechanisms of action has been increasingly recognized as more and more miRNA mutant mice are generated and analyzed (Olive et al., 2015), but a comprehensive review of relevant literature is still missing.

Here we sought to summarize the relative contribution of translation repression and mRNA degradation to miRNA regulation of functional targets in miRNA mutant mice. We focused on miRNA target genes whose protein and mRNA levels were measured concurrently in primary cells or tissues from mutant mice with genetic ablation or transgenic expression of individual miRNA genes. This includes a total of 159 target genes from 77 miRNA mutant mice (Table S1; Zhao et al., 2005, 2007; Lu et al., 2007, 2009, 2014; van Rooij et al., 2007; Vigorito et al., 2007; Dorsett et al., 2008; Liu et al., 2008, 2011, 2012, 2014; Wang et al., 2008, 2013a,b, 2014, 2015a,b; Boettger et al., 2009; Callis et al., 2009; O'Connell et al., 2009, 2010; Poy et al., 2009; Shan et al., 2009; Williams et al., 2009; Xin et al., 2009; Miyaki et al., 2010; Patrick et al., 2010; Yu et al., 2010; Biton et al., 2011; Boldin et al., 2011; Dunand-Sauthier et al., 2011; Jiang et al., 2011; Jordan et al., 2011; Ma et al., 2011, 2013; Nakamura et al., 2011; Sanuki et al., 2011; Shibata et al., 2011; Aurora et al., 2012; Callegari et al., 2012; Caruso et al., 2012; Dong et al., 2012; Gurha et al., 2012; Horie et al., 2012, 2013; Hsu et al., 2012; Liang et al., 2012, 2015; Mori et al., 2012; Tsai et al., 2012; Ucar et al., 2012; Wei et al., 2012, 2014; Zhuang et al., 2012; Belkaya et al., 2013; Bian et al., 2013; Danielson et al., 2013; Dorhoi et al., 2013; Dudda et al., 2013; Gebeshuber et al., 2013; Guo et al., 2013; Hasuwa et al., 2013; Heidersbach et al., 2013; Henao-Mejia et al., 2013; Khan et al., 2013; Mok et al., 2013; Song et al., 2013, 2014; Stadthagen et al., 2013; Tan et al., 2013; Wystub et al., 2013; Agudo et al., 2014; Ahmed et al., 2014; Burger et al., 2014; Chapnik et al., 2014; Dahan et al., 2014; Escobar et al., 2014; Giusti et al., 2014; Hu et al., 2014; Krzeszinski et al., 2014; Latreille et al., 2014; Pan et al., 2014; Stickel et al., 2014; Cushing et al., 2015; Jin et al., 2015; Kosaka et al., 2015; Kramer et al., 2015; Li et al., 2015a,b,c; Parchem et al., 2015; Sullivan et al., 2015; Sun et al., 2015; Tung et al., 2015; Xu et al., 2015; Yan et al., 2015; Zhang et al., 2015). Our analysis showed that 48% target genes are predominantly regulated by translation repression (76/159), 29% are regulated mainly by mRNA degradation (46/159), and 23% are regulated by both (37/159) (Figure 1). It is still unclear what determines the dominant mode of miRNA mechanism of action. As most of these studies measured target gene mRNA and protein levels under steady-state conditions, we speculate that differences in miRNA mechanism of action are not solely determined by the expression kinetics of miRNA or target mRNAs (Bazzini et al., 2012; Béthune et al., 2012; Djuranovic et al., 2012), but are instead attributed to cell type-, target mRNA-, or even miRNA-specific factors. Interestingly, almost all target genes identified in developing cells or tissues are mainly regulated by mRNA degradation, such as day 0 or day 2.5 cardiac cells (Heidersbach et al., 2013; Wei et al., 2014), embryonic stem cell-derived neurons (Tung et al., 2015), thymocytes (Belkaya et al., 2013; Henao-Mejia et al., 2013; Burger et al., 2014), bone marrow cells (Song et al., 2013), embryonic heart (Wystub et al., 2013; Liang et al., 2015), embryonic yolk sac (Wang et al., 2008), embryonic and neonatal epithelium (Ahmed et al., 2014), and fetal liver (Patrick et al., 2010). This is in sharp contrast to target genes identified in terminally differentiated cells, which are predominantly regulated by translation repression. It is conceivable that mRNA degradation gets rid of target gene mRNAs in a non-reversible way and provides an efficient way for cell fate determination, while translation repression is immediate, transient, and reversible, which is more suitable for differentiated cells to respond to environmental stresses. Our analysis also suggests miRNA-specific functional consequences. Several groups independently observed that target genes of miR-17~92 (Lu et al., 2007; Shan et al., 2009; Jiang et al., 2011; Bian et al., 2013; Danielson et al., 2013; Jin et al., 2015), miR-214 (Aurora et al., 2012; Wang et al., 2013b; Li et al., 2015b), miR-143/145 (Boettger et al., 2009; Jordan et al., 2011; Caruso et al., 2012; Dahan et al., 2014), and miR-146 (Boldin et al., 2011; Guo et al., 2013; Stickel et al., 2014) tend to be regulated at the translational level, but target genes of miR-122 (Hsu et al., 2012; Tsai et al., 2012), miR-140 (Miyaki et al., 2010; Nakamura et al., 2011) and miR-142 (Chapnik et al., 2014; Kramer et al., 2015; Sun et al., 2015) are often regulated by mRNA degradation. Interestingly, among miR-155 target genes, some are predominantly regulated by translation repression, some are mainly regulated by mRNA degradation, while the others are regulated by both mechanisms (Vigorito et al., 2007; Dorsett et al., 2008; Lu et al., 2009, 2014; O'Connell et al., 2009, 2010; Dudda et al., 2013; Escobar et al., 2014; Hu et al., 2014; Jin et al., 2015; Wang et al., 2015a), suggesting that different target genes of the same miRNA can be regulated through different mechanisms even in the same cell. It is a tempting possibility that *cis*-elements in mature miRNAs and target mRNAs determine the mechanism of miRNA action. Future investigation is warranted to identify these *cis*-elements, if they exist at all.

**Figure 1:**
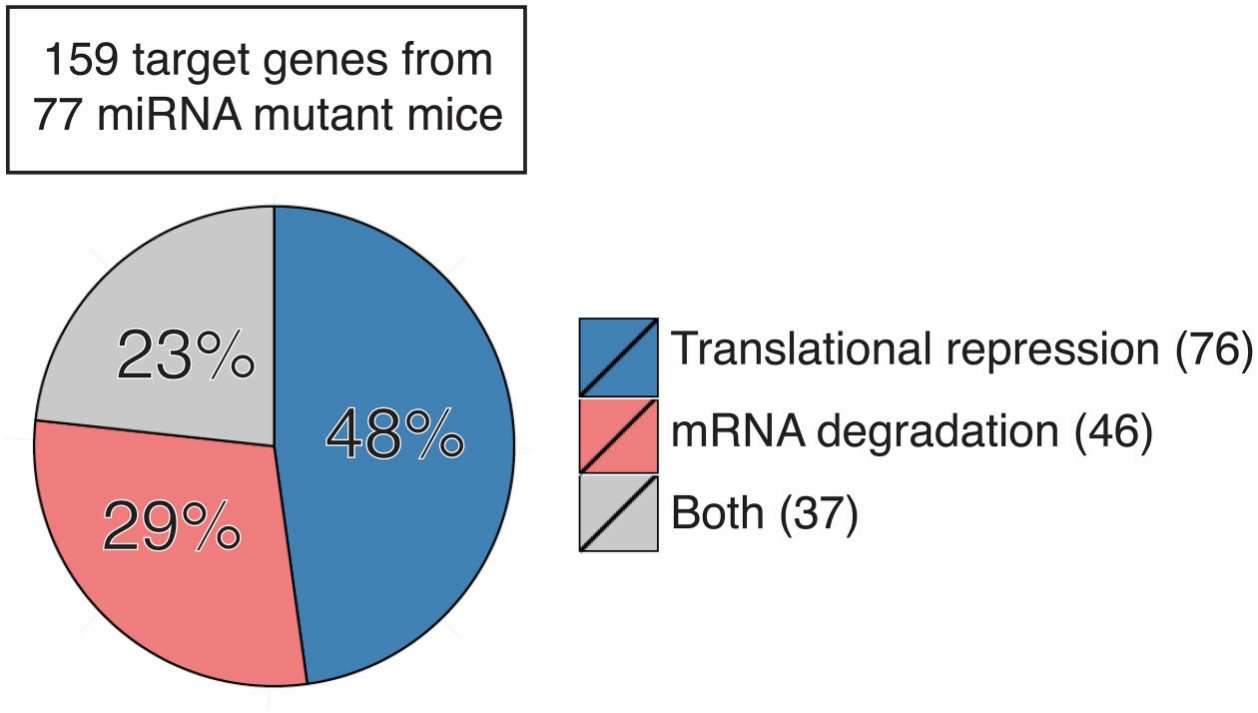
**Contribution of translation repression and mRNA degradation to miRNA regulation of target gene expression in primary cells and tissues from miRNA mutant mice**. Number in parentheses indicates the number of genes in each category. See also Table S1 for detailed information.

From a practical standpoint, measuring target gene protein levels is preferred to mRNA levels for the purpose of studying the effect of a miRNA on its target genes. Even for target genes predominantly regulated by mRNA degradation, the miRNA effect can still be captured by measuring their protein abundance. In the same vein, translatome analysis is more appropriate for measuring the global effect of a miRNA on its target genes, while transcriptome analysis often failed to identify any significant effect of miRNA deletion on its target genes, despite the obvious functional consequences in mutant mice (Matkovich et al., 2010; Boldin et al., 2011; Jiang et al., 2011; Agudo et al., 2014; Sullivan et al., 2015; Yuan et al., 2015). In the broader context of gene expression regulation, accumulating evidence shows that proteome and transcriptome are not sufficiently correlated to act as proxies for each other (Payne, 2015). miRNA-mediated translation regulation may play an important role in the de-coupling of translatome from transcriptome. We speculate that miRNAs emerged during evolution to increase the complexity of gene regulation, thereby contributing to the diversity of organisms.

## Conflict of interest statement

The authors declare that the research was conducted in the absence of any commercial or financial relationships that could be construed as a potential conflict of interest.
