# Supplementary material for: MicroRNA Mechanisms of Action: What have We Learned from Mice?
Source: Front Genet. 2015 Nov 16;6:328. doi: 10.3389/fgene.2015.00328 (PMC4644800; doi:10.3389/fgene.2015.00328)
Supplement: Supplementary file 1 [file Table1.PDF]

Table S1. Contribution of translation repression and mRNA degradation to miRNA regulation of target gene expression in primary cells and tissues from miRNA muntant mice

Citation: Jin HY and Xiao C (2015) MicroRNA mechanism of action: what have we learhed from mice? Front. Genet. 6:328. doi: 10.3389/fgene.2015.00328

miRNA	Validated target gene	Mutant mouse	Effect on mRNA	Effect on protein	Major miRNA mode of action	Cell or tissue type	Reference
miR-1	Hand2	miR-1-2 -/-	No change (qRT-PCR)	4-fold ↑ (WB)	Translational repression	Cardiac cells	Zhao et al., 2007
miR-1	Irx5	miR-1-2 -/-	1.7-fold ↑(qRT-PCR)	5-fold ↑ (WB)	Translational repression	Cardiac cells	Zhao et al., 2007
miR-1	Hand2	miR-1 TG	No change (RT-PCR)	~3 fold ↓ (WB)	Translational repression	Cardiac cells	Zhao et al., 2005
miR-1	Errβ	miR-1-1-/-;miR-1-2-/- (miR-1dKO)	2-fold ↑(qRT-PCR)	2-fold ↑ (WB)	mRNA degradation	Cardiac cells (post-natal day 2.5)	Wei et al., 2014
miR-1	Telokin	miR-1-1-/-;miR-1-2-/- (miR-1dKO)	8-fold ↑(qRT-PCR)	Not detectable in WT but expressed in KO	Both	Cardiac cells (P0)	Heidersbach et al., 2013
miR-1/133	Myocd	miR-1-1-/-;miR-1-2-/-;miR-133a-2-/-;miR-133a-1-/- (miR-1/133dKO)	2-fold ↑(qRT-PCR)	3-fold ↑ (WB)	Both	Cardiac cells (Embryonic heary E10.5)	Wystub et al., 2013
miR-7	Snca	miR-7a2 fl/fl;Rip-cre	1.5-fold ↑(qRT-PCR)	1.88-fold ↑ (WB)	Both	Islets	Latreille et al., 2014
miR-7	Pkcb	miR-7a2 fl/fl;Rip-cre	1.4-fold ↑(qRT-PCR)	6.25-fold ↑ (WB)	Translational repression	Islets	Latreille et al., 2014
miR-7	Cspa	miR-7a2 fl/fl;Rip-cre	1.5-fold ↑(qRT-PCR)	4.25-fold ↑ (WB)	Translational repression	Islets	Latreille et al., 2014
miR-7	Cplx1/2	miR-7a2 fl/fl;Rip-cre	1.8-fold ↑(qRT-PCR)	3.58-fold ↑ (WB)	Both	Islets	Latreille et al., 2014
miR-7	Pfn2	miR-7a2 fl/fl;Rip-cre	1.5-fold ↑(qRT-PCR)	2.05-fold ↑ (WB)	Both	Islets	Latreille et al., 2014
miR-7	Zdhhc9	miR-7a2 fl/fl;Rip-cre	2-fold ↑(qRT-PCR)	2.17-fold ↑ (WB)	mRNA degradation	Islets	Latreille et al., 2014
miR-7	Basp1	miR-7a2 fl/fl;Rip-cre	1.7-fold ↑(qRT-PCR)	4.44-fold ↑ (WB)	Translational repression	Islets	Latreille et al., 2014
miR-9	Foxg1	miR-9-2/9-3 -/-	No change (qRT-PCR)	2-fold ↑ (WB)	Translational repression	Cerebral hemispheres	Shibata et al., 2011
miR-9	Meis2	miR-9-2/9-3 -/-	No change (In situ hybridization)	~5-fold ↑ (IHC)	Translational repression	Telencephalon	Shibata et al., 2011
miR-9	Gsh2	miR-9-2/9-3 -/-	No change (In situ hybridization)	1.7-fold ↑(WB)	Translational repression	Cerebral hemispheres	Shibata et al., 2011
miR-9	Rest	miR-9-sponge-TG; Nestin-cre	50% ↑(qRT-PCR)	50% ↑(WT)	mRNA degradation	Hippocampal neuron	Giusti et al., 2014
miR-10a	Klf4	miR-10a -/-	15% ↑(qRT-PCR)	2-fold ↑(IHC)	Translational repression	Intestine	Stadthagen et al., 2013
miR-15a/16-1	Myb	miR-15a/16-1 fl/fl; CD2-cre	16% ↑(Microarray), 40% ↑(qRT-PCR)	9.6-fold ↑ (WB)	Translational repression	NK cells	Sullivan et al., 2015
miR-17	Fibronectin	miR-17 TG	No change (RT-PCR)	Complete abolish (WB)	Translational repression	Heart, kidney, lung and liver	Shan et al., 2009
miR-17~92	Rbl2	Sftpc-miR-17~92 TG (lung-specific)	8% ↓ (Probe1) or 11% ↓ (Probe2) (Microarray)	~3 fold ↓ (IHC)	Translational repression	Lung	Lu et al., 2007
miR-17~92	Pten	miR-17~92 fl/fl;CD4-cre or miR-17~92 fl/fl;Lck-cre	19% ↑(Microarray), 50%↑(qRT-PCR)	42% ↑(WB)	mRNA degradation	T cells (CD4+ CD25-)	Jiang et al., 2011
miR-17~92	Tgfbr2	miR-17~92 fl/fl;CD4-cre or miR-17~92 fl/fl;Lck-cre	2%↓ (Probe1), 16%↓ (Probe2), 32%↓ (Probe3) (Microarray)	31% ↑(WB)	Translational repression	T cells (CD4+ CD25-)	Jiang et al., 2011
miR-17~92	Creb1	miR-17~92 fl/fl;CD4-cre or miR-17~92 fl/fl;Lck-cre	15%↑(Probe1), 9%↓ (Probe2), 4%↑(Probe3), 5%↑(Probe4)	46% ↑(WB)	Translational repression	T cells (CD4+ CD25-)	Jiang et al., 2011
miR-17~92	Pten	miR-17~92 fl/fl;Emx-cre	No change (qRT-PCR)	1.4-fold ↑(WB)	Translational repression	Cortex	Bian et al., 2013
miR-17~92	Tbr2	miR-17~92 fl/fl;Emx-cre	No change (qRT-PCR)	1.6-fold ↑(WB)	Translational repression	Cortex	Bian et al., 2013
miR-17~92	Pten	miR-17~92 TG;SM22a-cre	50% ↓ (qRT-PCR)	~5 fold ↓ (WB)	Translational repression	Cardiac cells	Danielson et al., 2013
miR-17~92	Cx43	miR-17~92 TG;SM22a-cre	30% ↓ (qRT-PCR)	>10-fold fold ↓ (WB)	Translational repression	Cardiac cells	Danielson et al., 2013
miR-17~92	Pten	miR-17~92 fl/fl;Gzmb-cre	No change (qRT-PCR)	20% ↑(FACS)	Translational repression	LCMV inf CD8+ T cells	Khan et al., 2013
miR-17~92	Pten	miR-17~92 TG;Gzmb-cre	~30% ↓(qRT-PCR)	50% ↓(FACS)	Both	LCMV inf CD8+ T cells	Khan et al., 2013
miR-17~92	Ikzf4	miR-17~92 fl/fl;CD4-cre	1.73-fold ↑ (qPCR-array)	~3-fold ↑ (WB)	Both	In vitro differentiated Th17 cells	Liu et al., 2014
miR-17~92	Bim	miR-17~92 fl/fl;CD19-cre	No change (qRT-PCR)	1.6-fold ↑ (WB)	Translational repression	Naive B cells (IgM+ B220+)	Xu et al., 2015
miR-17~92	S1pr1	miR-17~92 fl/fl;CD19-cre	30% ↑ (qRT-PCR)	Slignt increase (FACS)	mRNA degradation	Naive B cells (IgM+ B220+)	Xu et al., 2015
miR-17~92	S1pr1	miR-17~92 fl/fl;CD19-cre	2-fold ↑ (qRT-PCR)	~10 fold ↑ (FACS)	Translational repression	Plasmablasts (CD138+ B220low)	Xu et al., 2015
miR-17~92	Ikzf1 (IKAROS)	miR-17~92 fl/fl;CD19-cre	1.8-fold ↑ (qRT-PCR)	1.8-fold ↑ (WB)	mRNA degradation	Activated B cells (LPS + IFN-gamma, day2)	Xu et al., 2015
miR-17~92	Pten	miR-17~92 fl/fl;Olig2-cre or miR-17~92-/-	2.3-fold ↑ (qRT-PCR)	1.7-fold ↑ (WB)	mRNA degradation	ESC derived motor neurons	Tung et al., 2015
miR-17~92	Ndfip1	miR-17~92 fl/fl;Olig2-cre or miR-17~92-/-	1.8-fold ↑ (qRT-PCR)	1.54-fold ↑ (WB)	mRNA degradation	ESC derived motor neurons	Tung et al., 2015
miR-17~92	Nedd4-2	miR-17~92 fl/fl;Olig2-cre or miR-17~92-/-	1.8-fold ↑ (qRT-PCR)	1.22-fold ↑ (WB)	mRNA degradation	ESC derived motor neurons	Tung et al., 2015
miR-17~92	Phlpp2	miR-17~92 TG;CD19-cre	No change (qRT-PCR)	3 fold ↓ (WB)	Translational repression	Naive B cells (CD5, CD43, and CD93 depleted)	Jin et al., 2015
miR-17~92	Pten	miR-17~92 TG;CD19-cre	No change (qRT-PCR)	25% ↓(WB)	Translational repression	Naive B cells (CD5, CD43, and CD93 depleted)	Jin et al., 2015

miR-17~92	Bim	miR-17~92 TG;CD19-cre	No change (qRT-PCR)	25% ↓(WB)	Translational repression	Naive B cells (CD5, CD43, and CD93 depleted)	Jin et al., 2015
miR-21	Spri1	miR-21 -/-	~10% ↓(Microarray, 25.5h)	1.5-fold ↑ (FACS)	Translational repression	Keratinocytes	Ma et al., 2011
miR-22	Purb	miR-22 -/-	3.5-fold ↑ (qRT-PCR)	2-fold ↑ (WB)	mRNA degradation	Cardiac cells	Gurha et al., 2012
miR-22	Tet2	miR-22 TG; Mx1-cre	65%↓ (qRT-PCR)	Complete abolish (WB)	Both	Bone marrow from plpC administrated mice	Song et al., 2013
miR-29	Igf1	miR-29a/b1 TG	30%↓ (qRT-PCR)	24% ↓(WB)	mRNA degradation	Epidydymis	Ma et al., 2013
miR-29	Klf4	miR-29a/b1-/-;miR-29b2/c-/-	2.1-fold ↑ (qRT-PCR)	1.8-fold ↑ (WB)	mRNA degradation	Lung	Cushing et al., 2015
miR-31	Gprc5a	miR-31 fl/fl;CD4-cre	4.19-fold ↑ (Microarray), 5-fold ↑ (qRT-PCR)	1.96-fold ↑ (WB)	mRNA degradation	Polarized iTreg cells	Zhang et al., 2015
miR-31	Ppp6c	miR-31 fl/fl;Keratin5-cre	3.5-fold ↑ (qRT-PCR)	3.7-fold ↑ (WB)	mRNA degradation	Epidermis from imiquimod treated mice	Yan et al., 2015
miR-33	Abca1	miR-33 -/- ; ApoE-/-	15% ↑ (qRT-PCR)	1.8-fold ↑ (WB)	Translational repression	Liver	Horie et al., 2012
miR-33	Crot	miR-33 -/- ; ApoE-/-	1.8-fold ↑ (qRT-PCR)	20% ↑ (WB)	mRNA degradation	Liver	Horie et al., 2012
miR-33	Abca1	miR-33 -/- ; ApoE-/-	2.2-fold ↑ (qRT-PCR)	~2-fold ↑ (WB)	mRNA degradation	Macrophage	Horie et al., 2012
miR-33	Abcg1	miR-33 -/- ; ApoE-/-	1.5-fold ↑ (qRT-PCR)	~2-fold ↑ (WB)	Both	Macrophage	Horie et al., 2012
miR-33	Nrip1 (RIP140)	miR-33 -/- ; ApoE-/-	1.6-fold ↑ (qRT-PCR)	1.5-fold ↑ (WB)	mRNA degradation	Macrophage	Horie et al., 2012
miR-33	Srebf1 (SREBP-1)	miR-33 -/-	1.7-fold ↑ (qRT-PCR)	~3-fold ↑ (WB)	Both	Hepatocytes	Horie et al., 2013
miR-34	Ccnd1 (Cyclind D1)	miR34b/c fl/fl; α1(I)-cre	20% ↑ (qRT-PCR, not significant)	2-fold ↑ (WB) or 5.16-fold ↑ (WB)	Translational repression	Osteoblast	Wei et al., 2012
miR-34	Satb2	miR34b/c fl/fl; α1(I)-cre	10% ↑ (qRT-PCR, not significant)	2-fold ↑ (WB)	Translational repression	Osteoblast	Wei et al., 2012
miR-34/449	Cp110	miR-34/449 -/- (miR-34a-/-;miR-34bc/-/-;miR-449abc-/-)	50% ↑ (qRT-PCR)	90% ↑ (WB)	Both	Tracheal Epithelia	Song et al., 2014
miR-34	Tgif2	miR-34a TG	>10-fold↓ (qRT-PCR)	~5-fold↓ (WB)	mRNA degradation	Osteoblast	Krzeszinski et al., 2014
miR-122	Cidec	miR-122fl/fl;Alb-cre	~7-fold ↑ (qRT-PCR, Microarray)	~2-fold ↑ (WB)	mRNA degradation	Liver	Hsu et al., 2012
miR-122	Mapre1	miR-122fl/fl;Alb-cre	~2-fold ↑ (qRT-PCR, Microarray)	~3-fold ↑ (WB)	Both	Liver	Hsu et al., 2012
miR-122	Adam10	miR-122fl/fl;Alb-cre	1.4-fold ↑ (qRT-PCR)	~2-fold ↑ (WB)	Both	Liver	Hsu et al., 2012
miR-122	Pparβ/δ	miR-122fl/fl;Alb-cre	8.3-fold ↑ (qRT-PCR)	>10-fold ↑ (WB)	mRNA degradation	Liver	Hsu et al., 2012
miR-122	Smarcd1	miR-122fl/fl;Alb-cre	3.8-fold ↑ (qRT-PCR)	~2-fold ↑ (WB)	mRNA degradation	Liver	Hsu et al., 2012
miR-122	Klf6	miR-122a -/-	1.8-fold ↑ (Microarray)	1.28-fold ↑ (WB)	mRNA degradation	Liver	Tsai et al., 2012
miR-124a	Lhx2	Rncr3-/- (miR-124a precursor KO)	No change (Luciferase Assay qRT-PCR)	2-fold ↑ (WB)	Translational repression	Retina	Sanuki et al., 2011
miR-126	Spred1	miR-126a -/-	2-fold ↑ (Microarray)	Not detectable in WT but expressed in KO	Both	E15.5 yolk sac	Wang et al., 2008
miR-126	Tsc1	miR-126a -/-	16% ↓(Microarray)	~3 fold ↑ (WB)	Translational repression	Plasmacytoid DCs	Agudo et al., 2014
miR-126	Fzd7	miR-126a -/- (AE9a transduced background)	39% ↑ (Microarray, P=0.17)	>10 fold ↑ (WB)	Translational repression	AML cells from quaternary BMT recipients	Li et al., 2015c
miR-128-2	Tarpp	miR-128-2fl/fl;Drd1a-cre	40% ↑(qRT-PCR)	2-fold ↑ (WB)	Both	Striatum	Tan et al., 2013
miR-133	Ccnd2	miR-133a-1-/-;miR-133a-2-/- (miR-133a-dKO)	1.7-fold ↑ (qRT-PCR)	2.3-fold ↑ (WB)	Both	Heart	Liu et al., 2008
miR-133	Dnm2	miR-133a-1-/-;miR-133a-2-/- (miR-133a-dKO)	2-fold ↑ (qRT-PCR)	7.2-fold ↑ (WB)	Translational repression	Tibialis anterior (TA) muscle	Liu et al., 2011
miR-137	Mitf	miR-137 TG	No change (Microarray), 10% ↓(qRT-PCR, not significant)	~2-fold ↓(WB and immunoflorescence)	Translational repression	Skin cells from tail	Dong et al., 2012
miR-140	Adamts5	miR-140-/-	1.8-fold ↑ (qRT-PCR)	~1.6-fold ↑ (Based on adamts5 positive cell	mRNA degradation	Chondrocyte	Miyaki et al., 2010
miR-140	Dnpep	miR-140-/-	1.8-fold ↑ (qRT-PCR)	~1.5-fold ↑ (WB)	mRNA degradation	Chondrocyte	Nakamura et al., 2011
miR-142	Wasl	miR-142-/-	3-fold ↑ (qRT-PCR)	2.2-fold ↑ (WB)	mRNA degradation	Megakaryocytes	Chapnik et al., 2014
miR-142	Cofilin2	miR-142-/-	8-fold ↑ (qRT-PCR)	3-fold ↑ (WB)	mRNA degradation	Megakaryocytes	Chapnik et al., 2014
miR-142	Tnfrsf13c (BAFF-R)	miR-142-/-	1.5-fold ↑ (qRT-PCR and Microarray)	1.5-fold ↑ (FACS)	mRNA degradation	B cells	Kramer et al., 2015
miR-142	IL-6	miR-142-/-	1.4-fold ↑ (Microarray)	3-fold ↑ (Elisa)	Both	Dendritic cells	Sun et al., 2015
miR-142	E2f7	miR-142-/-	9.9-fold ↑ (Microarray) or 10-fold ↑ (qRT-PCR)	Not detectable in WT but expressed in KO	Both	T cells	Sun et al., 2015
miR-142	E2f8	miR-142-/-	7.6-fold ↑ (Microarray) or 15-fold ↑ (qRT-PCR)	Not detectable in WT but expressed in KO	Both	T cells	Sun et al., 2015
miR-143/145	Ace-1	miR-143/145 -/-	No change (Microarray)	4.9-fold ↑ (SILAC), 4.3-fold ↑ (WB)	Translational repression	Aorta cells	Boettger et al., 2009
miR-143/145	Ace-1	miR-143/145 -/-	2.1-fold ↑ (qRT-PCR)	6.5-fold ↑ (WB)	Translational repression	Aorta cells	Dahan et al., 2014
miR-143/145	Klf5	miR-143/145 -/-	1.4-fold ↑ (qRT-PCR)	1.5-fold ↑ (WB)	mRNA degradation	Aorta cells	Xin et al., 2009
miR-143/145	Orp8	miR-143/145 -/-	20% ↑ (qRT-PCR)	~1.8 -fold ↑ (SILAC, WB)	Translational repression	Liver cells	Jordan et al., 2011
miR-143/145	Orp8	miR-143 Dox TG	No change (qRT-PCR)	2-fold↓ (SILAC, WB)	Translational repression	Liver cells	Jordan et al., 2011

miR-145	Klf4	miR-145 -/-	30% ↑ (qRT-PCR)	40% ↑ (WB)	mRNA degradation	Lung (hypoxia, day 14)	Caruso et al., 2012
miR-145	Kfl5	miR-145 -/-	10% ↑ (qRT-PCR)	5-fold ↑ (WB)	Translational repression	Lung (hypoxia, day 14)	Caruso et al., 2012
miR-146a	Irak1	miR-146a -/-	No change (qRT-PCR)	1.6-fold ↑ (WB)	Translational repression	BM-derived macrophages	Boldin et al., 2011
miR-146a	Irak1	miR-146a -/-	No change (qRT-PCR)	2.3-fold ↑ (WB)	Translational repression	B cells	Boldin et al., 2011
miR-146a	Traf6	miR-146a -/-	No change (qRT-PCR)	26-fold ↑ (WB)	Translational repression	BM-derived macrophages	Boldin et al., 2011
miR-146a	Traf6	miR-146a -/-	No change (qRT-PCR)	3.5-fold ↑ (WB)	Translational repression	B cells	Boldin et al., 2011
miR-146a	Traf6	miR-146a -/-	54% ↑ (Microarray, stimulated with BMDC)	2.5-fold ↑ (WB, purified from allo-HTC recipient)	Both	CD4+CD8+ T cells	Stickel et al., 2014
miR-146a	Fas	miR-146a TG	No change (Microarray)	2-fold↓ (Facs)	Translational repression	Germinal center B cells	Guo et al., 2013
miR-146a/b	Traf6	miR-146a or 146b TG (T cell specific)	60% ↓(miR-146a TG) and 40% ↓ (miR-146b TG) (qRT-PCR)	~90% both in miR-146a TG and miR-146b TG (WB)	Both	Thymocytes	Burger et al., 2014
miR-153	App	miR-153 TG	10%↑ (qRT-PCR)	26% ↓ (WB)	Translational repression	Cerebral cortex	Liang et al., 2012
miR-153	Aplp2	miR-153 TG	20%↑ (qRT-PCR)	39% ↓ (WB)	Translational repression	Cerebral cortex	Liang et al., 2012
miR-155	Ship1	miR-155 -/-	1.8-fold↑ (LPS, 24h qRT-PCR)	3 fold ↑ (LPS, 24h, WB)	Both	Activated BMDM	O'Connell et al., 2009
miR-155	Ship1	miR-155 -/-	~25% ↑ (qRT-PCR)	~50% ↑ (WB)	Both	Dendritic cells	O'Connell et al., 2010
miR-155	Socs1	miR-155 -/-	~10% ↑ (qRT-PCR)	~2 fold ↑ (WB)	Translational repression	Dendritic cells	O'Connell et al., 2010
miR-155	Socs1	miR-155 -/-	No change (qRT-PCR)	5-fold ↑ (WB)	Translational repression	Treg cells	Lu et al., 2009
miR-155	Socs1	miR-155 -/-	1.6-fold ↑ (qRT-PCR)	~2-fold ↑ (WB)	Both	CD8+ T cells	Dudda et al., 2013
miR-155	Aicda (AID)	miR-155 -/-	~50% ↑ (day 3, qRT-PCR)	~3 fold ↑ (day 3, WB)	Both	Activated B cells	Dorsett et al., 2008
miR-155	Sfpi1 (PU.1)	miR-155 -/-	1.47-fold ↑ (day1,Microarray), 2.8-fold ↑ (qRT-PCR)	Not detectable in WT but expressed in KO (WB)	Both	Activated B cells	Vigorito et al., 2007
miR-155	Sfpi1 (PU.1)	miR-155 -/-	2.2-fold ↑ (day 4, qRT-PCR)	2-fold ↑ (day 4, WB)	mRNA degradation	Activated B cells	Lu et al., 2014
miR-155	c-Fos	miR-155 -/-	1.5-fold ↑ (Unstim), 2.8-fold ↑ (24h LPS) (qRT-PCR)	25-fold ↑ (WB)	Translational repression	BM-derived DC	Dunand-Sauthier, et al.,
miR-155	Jarid2	miR-155 -/-	1.9-fold ↑ (qRT-PCR)	2-fold ↑ (WB)	mRNA degradation	Th17 cells	Escobar et al., 2014
miR-155	c-Maf	miR-155 -/-	1.6-fold ↑ (RNA-seq)	2.4-fold ↑ (WB)	Both	Th17 cells	Escobar et al., 2014
miR-155	Peli1	miR-155 -/-	1.3-fold ↑ (RNA-seq)	1.7-fold ↑ (WB)	Both	CD4+ T cells	Hu et al., 2014
miR-155	Ikbke	miR-155 -/-	1.4-fold ↑ (RNA-seq)	2-fold ↑ (WB)	Both	CD4+ T cells	Hu et al., 2014
miR-155	Fosl2	miR-155 -/-	1.3-fold ↑ (RNA-seq)	2.8-fold ↑ (WB)	Translational repression	CD4+ T cells	Hu et al., 2014
miR-155	Hif-1α	miR-155 -/-	No change (qRT-PCR)	~3 fold ↑ (WB)	Translational repression	Splanic MDSC	Wang et al., 2015
miR-155	Ship1	Eμ-miR-155 TG (B cell specific)	20% ↓ (Microrarray, P= 0.12)	2-fold ↓ (WB, P < 0.01)	Translational repression	Naive B cells	Jin et al., 2015
miR-181	Pten	miR-181a1/b1 -/-	65% ↑ (RNA-seq)	~2 fold ↑ (WB)	Both	DP Thymocytes	Henao-Mejia et al., 2013
miR-185	Mzb1	miR-185 TG	90% ↓(Microarray and qRT-PCR)	Complete abolish (WB)	mRNA degradation	DN3 Thymocytes	Belkaya et al., 2013
miR-185	NFATc3	miR-185 TG	80% ↓(Microarray and qRT-PCR)	~80%↓ (WB)	mRNA degradation	DN3 Thymocytes	Belkaya et al., 2013
miR-188	Rictor	miR-188 -/-	No change (Microarray) & No change upon anti-miR treatment	~2 fold ↑ in 3mo-old, and >10-fold in 18mo-old mice, (WB)	Translational repression	Bone marrow mesenchymal stem cells	Li et al., 2015a
miR-188	Hdac9	miR-188 -/-	No change (Microarray) & No change upon anti-miR treatment	~2 fold ↑ in 3mo-old, and >10-fold in 18mo-old mice, (WB)	Translational repression	Bone marrow mesenchymal stem cells	Li et al., 2015a
miR-193	Wt1	miR-193 TG (Dox-inducible)	30% ↓ in day 4, 30% ↓ in day 8 of Dox-treatment (qRT-PCR)	>3-fold↓ in day 4, >10-fold ↓ in day 8 of Dox-treat (WB)	Translational repression	Glomerulus	Gebeshuber et al., 2013
miR-196	Hoxc8	αP2-miR-196a TG (fat specific)	No change (qRT-PCR, WAT-progenitor cells day 6&12)	2-fold ↓ (WB)	Translational repression	Inguinal WAT, epididymal WAT and intescapular BAT	Mori et al., 2012
miR-200/429	Zeb1	miR-200-/-;429-/-	5% ↓(qRT-PCR)	~3 fold ↑ (WB)	Translational repression	Pituitary tissue	Hasuwa et al., 2013
miR-204	Jarid2	miR-204 TG	30% ↓(qRT-PCR)	30%↓ (WB)	mRNA degradation	Heart from embryo	Liang et al., 2015
miR-205	Phlda3	miR-205fl/fl;EIIa-cre	24% ↑ (Microarray)	51% ↑ (WB)	Both	Hair follicle stem cells	Wang et al., 2013a
miR-205	Inppl1	miR-205fl/fl;EIIa-cre	28% ↑ (Microarray)	42% ↑ (WB)	Both	Hair follicle stem cells	Wang et al., 2013a
miR-206	Hdac4	miR-206-/-	No change (qRT-PCR)	2.9-fold ↑ (WB)	Translational repression	Skeletal muscle	Williams et al., 2009
miR-206	Pax7	miR-206-/-	2.5-fold ↑ (qRT-PCR, Differentiaion media, 5days)	Not detectable in WT but expressed in KO	Both	Satellite cells	Liu et al., 2012
miR-206	Notch3	miR-206-/-	1.8-fold ↑ (qRT-PCR, Differentiaion media, 5days)	~2-fold ↑ (WB, Differentiaion media, 5days)	mRNA degradation	Satellite cells	Liu et al., 2012
miR-206	Igfbp5	miR-206-/-	1.3-fold ↑ (qRT-PCR, Differentiaion media, 5days)	~3-fold ↑ (WB, Differentiaion media, 5days)	Translational repression	Satellite cells	Liu et al., 2012
miR-208	Thrap1	miR-208-/-	No change (RT-PCR)	~2-fold↑ (WB)	Translational repression	Cardiac cells	van Rooij et al., 2007
miR-208a	Thrap1	miR-208a-/-	No change (RT-PCR)	50%↑ (WB)	Translational repression	Cardiac cells	Callis et al., 2009
miR-208a	Myostatin	miR-208a-/-	No change (RT-PCR)	50%↑ (WB)	Translational repression	Cardiac cells	Callis et al., 2009

miR-208a	Thrap1	miR-208a TG	No change (RT-PCR)	30%↓ (WB)	Translational repression	Cardiac cells	Callis et al., 2009
miR-208a	Myostatin	miR-208a TG	No change (RT-PCR)	50%↓ (WB)	Translational repression	Cardiac cells	Callis et al., 2009
miR-210	CD23 (Fcer2a)	miR-210 TG	11.7-fold ↓ (Microarray)	50-fold ↓ (Facs)	Both	B cells	Mok et al., 2013
miR-210	Hif-1α	miR-210fl/fl;CD4-cre	4-fold ↑ (qRT-PCR)	~2-fold ↑ (WB)	mRNA degradation	In vitro differentiated Th17 cells	Wang et al., 2014
miR-212/132	FoxO3a	αMHC-miR-212/132, (cardiomyocyte-specific)	25% ↓(qRT-PCR, P=0.08)	40% ↓(WB, P<0.005)	Both	Cardiac cells	Ucar et al., 2012
miR-214	Ncx1	miR-214-/-	No change (Microarray)	1.6-fold ↑ (WB)	Translational repression	Cardiac cells	Aurora et al., 2012
miR-214	Bim	miR-214-/-	No change (Microarray)	1.8-fold ↑ (WB)	Translational repression	Cardiac cells	Aurora et al., 2012
miR-214	CamkIId	miR-214-/-	No change (Microarray)	1.5-fold ↑ (WB)	Translational repression	Cardiac cells	Aurora et al., 2012
miR-214	Ppif	miR-214-/-	No change (Microarray)	1.5-fold ↑ (WB)	Translational repression	Cardiac cells	Aurora et al., 2012
miR-214	Atf4	bglap2-miR-214 TG (Osteoblast-specific)	No change (Luciferase Assay qRT-PCR)	~3-fold↓ (WB)	Translational repression	Intraosseous cells	Wang et al., 2012
miR-214	Ctnnb1 (β-catenin)	K14-rtTA-miR-214TG (Dox-inducible)	40% ↓ (Microarray and qRT-PCR)	40% ↓ (WB) or 50%↓ (Immunoflorescence)	mRNA degradation	Embryonic (E17.5) or neonetal (P2.5) epithelium	Amed et al., 2014
miR-214	Atf4	miR-214 TG;Alb-cre (Liver specific)	No change (qRT-PCR)	20%↓ (WB)	Translational repression	Liver cells	Li et al., 2015b
miR-221	Cdkn1b (p27)	α1-AT-miR-221 TG (Liver specific)	No change (Microarray)	4-fold↓ (WB)	Translational repression	Liver cells	Callegari et al., 2012
miR-221	Bmf	α1-AT-miR-221 TG (Liver specific)	No change (Microarray)	2.5-fold↓ (WB)	Translational repression	Liver cells	Callegari et al., 2012
miR-223	Pknox1	miR-223-/-	1.8-fold ↑ (qRT-PCR, LPS) or No change (qRT-PCR, IL-4)	3-fold ↑ (WB)	Both	BM-derived macrophages or Adipose	Zhuang et al., 2012
miR-223	Cxcl2	miR-223-/-	8-fold ↑ (Microarray, day 21)	~10-fold ↑ (Immunoassay)	mRNA degradation	Mtb challenged lung	Dorhoi et al., 2013
miR-223	Ccl3	miR-223-/-	4-fold ↑ (Microarray, day 21)	~1.8-fold ↑ (Immunoassay)	mRNA degradation	Mtb challenged lung	Dorhoi et al., 2013
miR-223	IL-6	miR-223-/-	3-fold ↑ (Microarray, day 21)	~7-fold ↑ (Immunoassay)	Both	Mtb challenged lung	Dorhoi et al., 2013
miR-302	Fgf15	miR-302-/-	2.5-fold ↑ (qRT-PCR, E9.5)	1.8-fold ↑ (WB, E11.5)	mRNA degradation	Brain from Embryo	Parchem et al., 2015
miR-361	Phb1	αMHC-miR-361 TG (cardiomyocyte-specific)	No change (qRT-PCR)	~2-fold ↓(WB)	Translational repression	Cardiac cells	Wang et al., 2015a
miR-375	Gphn	miR-375-/-	1.8-fold ↑ (qRT-PCR)	1.9-fold ↑ (WB)	mRNA degradation	Pancreatic islets	Poy et al., 2009
miR-375	HuD	miR-375-/-	3.5-fold ↑ (qRT-PCR)	3.3-fold ↑ (WB)	mRNA degradation	Pancreatic islets	Poy et al., 2009
miR-375	Cadm1	miR-375-/-	1.8-fold ↑ (qRT-PCR)	2.4-fold ↑ (WB)	mRNA degradation	Pancreatic islets	Poy et al., 2009
miR-375	Nnat	miR-375-/-	1.8-fold ↑ (qRT-PCR)	6.4-fold ↑ (WB)	Translational repression	Pancreatic islets	Poy et al., 2009
miR-375	Th	miR-375-/-	2.0-fold ↑ (qRT-PCR)	2.5-fold ↑ (WB)	mRNA degradation	Pancreatic islets	Poy et al., 2009
miR-375	Klf5	Dicer fl/fl; Villin-Cre ERT2 (iKO)	No change (qRT-PCR)	5.5-fold ↑ (IHC)	Translational repression	Colon	Biton et al., 2011
miR-378	Pde1b	miR-378 TG	60% ↓(qRT-PCR)	60% ↓(WB)	mRNA degradation	Brown fat (BAT)	Pan et al., 2014
miR-144/451	14-3-3z	miR-144/451 -/-	25% ↑ (Microarray)	20-fold ↑ (WB, from bone marrow)	Translational repression	Erythroblast from bone marrow or spleen	Yu et al., 2010
miR-451	14-3-3z	miR-451 -/-	3.0-fold ↑ (qRT-PCR), 8.6-fold ↑ (Microarray)	2.0-fold ↑ (WB)	mRNA degradation	E14.5 fetal liver TER119+ erythrocytes	Patrick et al., 2010

Methods:
WB, Western blot; IHC, Immunohistochemistry; FACS, Fluorescence-activated cell sorting; ELISA, Enzyme-linked immunosorbent assay; SILAC, Stable Isotopic Labeling using Amino Acids in Cell Culture.

Criteria used to determine major miRNA mode of action:
(1) Translation repression: the contribution of mRNA change to protein change is 20% or less;
(2) mRNA degradation: the contribution of mRNA change to protein change is 80% or more;
(3) Both: the contribution of mRNA change to protein change is in the range of 20-80%.
